# What can we learn about beat perception by comparing brain signals and stimulus envelopes?

**DOI:** 10.1371/journal.pone.0172454

**Published:** 2017-02-22

**Authors:** Molly J. Henry, Björn Herrmann, Jessica A. Grahn

**Affiliations:** Brain and Mind Institute, Department of Psychology The University of Western Ontario, London, ON, Canada; Australian Research Council Centre of Excellence in Cognition and its Disorders, AUSTRALIA

## Abstract

Entrainment of neural oscillations on multiple time scales is important for the perception of speech. Musical rhythms, and in particular the perception of a regular beat in musical rhythms, is also likely to rely on entrainment of neural oscillations. One recently proposed approach to studying beat perception in the context of neural entrainment and resonance (the “frequency-tagging” approach) has received an enthusiastic response from the scientific community. A specific version of the approach involves comparing frequency-domain representations of acoustic rhythm stimuli to the frequency-domain representations of neural responses to those rhythms (measured by electroencephalography, EEG). The relative amplitudes at specific EEG frequencies are compared to the relative amplitudes at the same stimulus frequencies, and enhancements at beat-related frequencies in the EEG signal are interpreted as reflecting an internal representation of the beat. Here, we show that frequency-domain representations of rhythms are sensitive to the acoustic features of the tones making up the rhythms (tone duration, onset/offset ramp duration); in fact, relative amplitudes at beat-related frequencies can be completely reversed by manipulating tone acoustics. Crucially, we show that changes to these acoustic tone features, and in turn changes to the frequency-domain representations of rhythms, do not affect beat perception. Instead, beat perception depends on the pattern of onsets (i.e., whether a rhythm has a simple or complex metrical structure). Moreover, we show that beat perception can differ for rhythms that have numerically identical frequency-domain representations. Thus, frequency-domain representations of rhythms are dissociable from beat perception. For this reason, we suggest caution in interpreting direct comparisons of rhythms and brain signals in the frequency domain. Instead, we suggest that combining EEG measurements of neural signals with creative behavioral paradigms is of more benefit to our understanding of beat perception.

## Introduction

Perception of temporal patterns is fundamental to normal hearing, speech, motor control, and music. Sensitivities to certain patterns are seemingly unique to humans [1–3], such as our sensitivity to musical rhythm, in which we rapidly identify the “beat”–a perceived pulse that marks isochronous points in time [4, 5], that we move to, and against which the timing of other events is measured. The sense of a beat emerges during listening to nonisochronous temporal patterns that do not contain explicit accents [6] as well as “syncopated” patterns, where the “on-beat” locations do not necessarily coincide with sound onsets [7, 8]. Thus, the beat must (in at least some cases) be internally generated through a process termed induction [9, 10].

Behaviorally, beat perception abilities are often measured using production tasks, where individuals make a repetitive motor response (e.g., tapping a finger) in time with the perceived beat in rhythmic stimuli [11–16]. Perceptual measures of beat perception involve making judgments about, for example, whether a single event deviates from isochrony [13], whether the overall tempo of a sequence has changed [14, 17], or whether a metronome superimposed onto a piece of music is on or off the beat [12, 15]. Unfortunately, these tasks are not suitable to answer questions regarding the age at which beat perception emerges during development [18], the rate with which beat perception deteriorates with some disorders [19], or the specificity of beat perception to humans [20, 21]. This is because special populations like infants, some patients, and non-human animals might not be capable of understanding task instructions, or may lack other cognitive or memory skills required to perform standard beat production or perception tasks. In addition, the nature of the task may actually alter the beat percept that would have occurred in the absence of the task (e.g., tapping to the beat provides haptic and kinesthetic feedback that interacts with perception of the beat induced by the stimulus alone [22]). Thus, an important ongoing scientific endeavor is to identify a clear and easily measured *neural* marker of beat perception that is independent of task performance.

In this regard, one intuitive explanation for beat perception arises from studies of neural oscillations, entrainment, and resonance [9, 23]. Briefly, neural oscillations reflect fluctuations in local neuronal excitability, meaning that they influence the likelihood of neuronal firing in a periodic fashion [24–28]. In the presence of rhythmic sensory input, the phase [29–32] or amplitude envelope [33, 34] of neural oscillations can become synchronized with the stimulus rhythm through *entrainment*. In the case of entrainment of a nonlinear oscillator by a stimulus rhythm, oscillations can emerge at frequencies that are not present in the stimulation through a phenomenon called *resonance*. The emergence of subharmonic oscillations through resonance has been hypothesized to give rise to beat perception [9].

A recently proposed approach to studying beat perception in the context of neural entrainment and resonance (the “frequency-tagging” approach) has received an enthusiastic response from the scientific community [35–40], as it has notably has the potential to deliver a neural marker of beat perception. Generally, the approach involves estimating the amplitude of the “steady-state evoked potential” at the beat rate by inspecting the frequency-domain representations of brain signals measured using electroencephalography (EEG). Previous work using this approach has demonstrated that asking participants to *imagine* accents on every second or every third event (giving rise to perception of a duple or triple meter, respectively) leads to amplitude increases at frequencies corresponding to the imagined meter (half or one-third of the event rate, respectively) [41]. Moreover, moving one’s body at half of the event rate (emphasizing a duple meter) leads to subsequent enhancements of the neural signal at that duple meter frequency during subsequent listening (without movement) [42].

The specific version of the frequency-tagging approach that is our focus here involves directly comparing frequency-domain representations of acoustic rhythms to frequency-domain representations of neural responses to those rhythms [43–45]. In this version of the approach, the amplitude envelopes of stimulus waveforms are obtained using the Hilbert transform. Then, the envelopes are submitted to a fast Fourier transform (FFT), which yields the frequency-domain representation of the rhythms’ temporal structure (i.e., the stimulus amplitude spectrum). The EEG data recorded during listening to the rhythm are also submitted to a FFT, yielding the neural amplitude spectrum. (We note that the transformation from the time domain using the FFT yields both amplitude [or power after squaring] and phase spectra in the frequency domain. For the purposes of the current discussion, we are concerned only with the amplitude information, and refer to frequency-domain representations interchangeably with amplitude spectra.) Finally, the two amplitude spectra are directly compared. When the (z-scored [44] or percent difference [45]) magnitude of the neural response at a particular “beat-related” frequency exceeds the (z-scored or percent difference) magnitude of the peak at the same frequency in the stimulus spectrum, the frequency is identified as “enhanced” in the neural response. The enhanced peaks at beat-related frequencies are proposed to reflect entrainment of a nonlinear oscillator by a stimulus rhythm and to provide empirical support for neural resonance as a neural correlate of beat perception [9].

Neural oscillations and entrainment have also been proposed to play an important mechanistic role in speech perception [46]. The relation between the fidelity of neural entrainment to speech and perception of that speech is commonly assessed by quantifying the degree of match between the (envelope of the) speech signal and the pattern of neural oscillations in, for example, the theta frequency band (4–8 Hz; [47] but see [48] for a note of caution). Although many authors do not make strong assumptions about how strictly the input (speech envelope) should match the output (brain responses to speech; [49]), others optimize the correspondence in a stimulus driven way [50, 51] or explicitly model transformations of the input based on knowledge of the peripheral [52] or central [53, 54] auditory system. In contrast to studies of speech-envelope tracking that are either agnostic to the nature of input–output transformation, or explicitly model the expected transformations performed by the auditory system, the version of the “frequency-tagging” approach to studying beat perception with which we are concerned [36, 44, 45] implicitly assumes that the brain signal (output) faithfully represents the stimulus envelope (input) and interprets deviations from this assumption as evidence of nonlinear resonance.

Here, however, we suggest that the presence of neural resonance cannot be inferred from comparing neural amplitude spectra to stimulus amplitude spectra (note that this argument is restricted to the implementation of the methodology–we are in no way questioning the validity of neural resonance theory, only the use of this specific version of the frequency-tagging approach to support the theory). The reason is that many alterations can be made to the stimulus envelope that will substantially alter the stimulus spectrum (e.g., alterations to tone duration, or onset/offset ramp duration). However, these alterations do *not* affect onset structure (i.e., the relative timings of event onsets) and thus are unlikely to affect beat perception. Thus, the nature of the relationship between the stimulus spectrum and perception is unclear.

The current paper demonstrates that direct frequency-domain comparisons of stimulus envelopes and brain signals in the context of beat perception are invalid in two ways. First, we analyzed previous rhythms that have been used to investigate the presence of neural resonance at beat-related frequencies during rhythm listening [44]. We systematically varied acoustic properties of the rhythms (tone duration and onset/offset ramp duration) to demonstrate that the relative amplitudes at beat-related frequencies in the stimulus spectra are highly sensitive to changes in the stimulus envelope. Second, we report behavioral data from two studies in which listeners rated the perceived beat salience of rhythms a) whose stimulus spectra varied despite no changes in the strength of the perceived beat, and b) whose stimulus spectra were identical despite differences in perceived beat strength. In sum, these experiments demonstrate that stimulus spectra and beat perception are dissociable, which we argue renders direct comparisons the EEG signal to the stimulus in the frequency domain is unlikely to be revealing with respect to the neural correlates of beat perception.

## Experiment 1

### Methods

**Fig 1:**
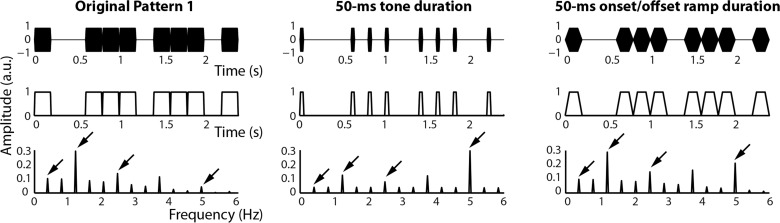
Examples of the acoustic manipulations applied to one representative rhythm analyzed in Experiment 1. The onset structure of the original rhythm (left column) was preserved. Tone duration (middle column) and onset/offset ramp duration (right column) were parametrically varied. After obtaining the amplitude envelopes (middle row) of the stimulus waveforms (top row) via a Hilbert transform, the envelopes were transformed to the stimulus spectra in the frequency domain using a FFT (bottom row). Arrows mark the beat-related frequencies 0.416 Hz (1:12), 1.25 Hz (1:4), 2.5 Hz (1:2), and 5 Hz (1:1).

**Fig 2:**
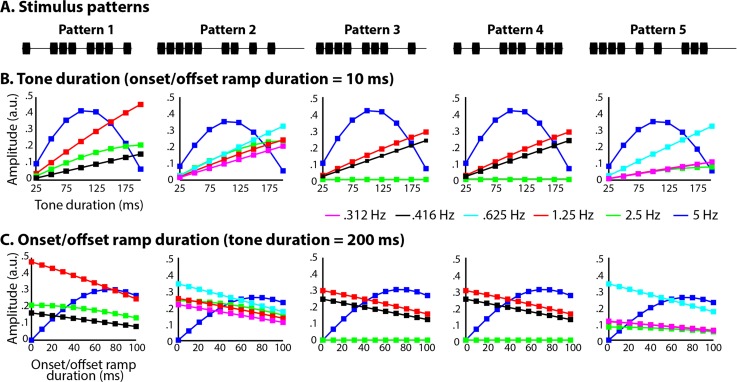
For each of the 5 stimulus patterns used in [44] (**A**), frequency-domain amplitudes at beat-related frequencies varied as a function of **B)** tone duration (onset/offset ramp duration was fixed at 10 ms) and **C)** onset/offset ramp duration (tone duration was fixed at 200 ms), but the functions were different for each frequency and each rhythm. See Supporting Information S1 Fig for all tested combinations of tone duration and onset/offset ramp duration for all stimulus patterns.

In order to demonstrate the effects of varying acoustic properties of rhythms on stimulus spectra, we analyzed five rhythms (referred to as Patterns 1–5), originally taken from [6] and also studied in [44]. Each pattern was generated using Matlab software (R2014b, Mathworks) at a sampling rate of 44,100 Hz from 990-Hz sine tones (as described in [44]). The base rate of the patterns was 5 Hz, meaning that all inter-onset intervals between tones were integer multiples of 200 ms. We parametrically varied tone duration and onset/offset ramp duration to assess how subtle changes in the time-domain representation of the stimulus alter its frequency-domain representation. Rhythms were composed of tones taking on one of the following durations: 25, 50, 75, 100, 125, 150, and 200 ms, and one of the following onset/offset ramp durations 0, 10, 20, 30, 40, 50, 60, 70, 80, 90, and 100 ms. For each tone duration, we only tested onset/offset ramp durations that were less than or equal to half of the tone duration. Examples of the acoustic manipulations are shown in Fig 1 for one of the rhythms. All five original stimulus patterns can be seen in Fig 2.

Each rhythm was looped in a repeating fashion so that the total stimulus duration was 33.6 s for Patterns 1, 3, and 4 and 35.2 s for Pattern 2 and 5. Given the assumption of cyclic data inbuilt into the FFT [55], we opted for durations that allowed an integer number of rhythm presentations. We obtained the amplitude envelopes for each rhythm as the modulus of the complex output of the Hilbert transform (Matlab function *hilbert*; Fig 1, middle row; we did not make use of the implementation of the Hilbert transform featured in the MIR toolbox as in [44] for reasons we will discuss below). Then, each amplitude envelope was submitted to a FFT and the stimulus spectrum was calculated as the modulus of the complex FFT output (Fig 1, third row).

For each rhythm, we evaluated how frequency-domain amplitudes at “beat- and meter-related frequencies” (as defined in [44]) changed with tone duration and onset/offset ramp duration. For Patterns 1, 3, and 4, we examined amplitudes at 0.416 Hz (1:12 relation to 5-Hz base rate), 1.25 Hz (1:4), 2.5 Hz (1:2) and 5 Hz (1:1, base rate). Musically, these are duple patterns. Thus, if 5 Hz is considered the ‘eighth-note’ duration, 2.5 Hz corresponds to a quarter-note duration, 1.25 Hz corresponds to a half-note duration, and .416 Hz corresponds to a whole-note duration. For Patterns 2 and 5, we examined amplitudes at 0.312 Hz (1:16), 0.625 Hz (1:8), 1.25 Hz (1:4), 2.5 Hz (1:2), and 5 Hz (1:1). Musically, these are triple patterns. Thus, if 5 Hz is considered the ‘eighth-note’ duration, 2.5 Hz corresponds to a quarter-note duration, 1.25 Hz corresponds to a half-note duration, 0.625 Hz corresponds to a dotted-half-note duration (one triple measure), and 0.312 Hz corresponds to two dotted-half-note durations (two triple measures).

**Fig 3:**
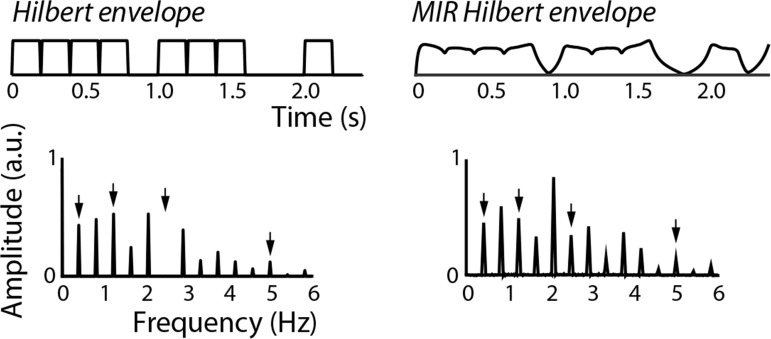
Comparison of envelopes (top) and stimulus spectra (bottom) obtained by using Matlab’s *Hilbert* function (left) or the MIR-implemented Hilbert transform, shown here for “Pattern 3” (from [44]). Since the MIR toolbox makes use of time-domain filtering, envelopes are smooth and frequency spectra differ from those obtained from the Matlab Hilbert transform. The most obvious discrepancy is at 2.5 Hz, where there is no energy in the spectrum obtained using the Matlab *Hilbert* function.

We also wanted to assess the consequences of using the MIR Toolbox implementation of the Hilbert transform which was used in by [44] versus the Matlab *hilbert* function to obtain the amplitude envelope of the stimulus. Of note, the MIR Toolbox documentation cautions against making use of their built-in Hilbert implementation. Upon inspection, the MIR Toolbox applies a time-domain filter that yields smoothed stimulus envelopes (Fig 3). Importantly, the nature of the filtering (an infinite-impulse response, IIR, filter with a low-pass cutoff of 50 Hz) is not based on assumptions about the operations performed by the peripheral or central auditory system. Since the envelopes coming from the Matlab *hilbert* function and the MIR-implemented Hilbert transform are different, their amplitude spectra are different. Thus, the smoothing produces frequency-domain representations of stimulus envelope that are not in agreement with the standard Hilbert implementation. To assess the degree to which these differences may alter the results reported in [44], we calculated the stimulus amplitude spectra for each rhythm using two processing pipelines: one with the MIR-implemented Hilbert transform and the other with the Matlab *hilbert* function. Then, for each rhythm, we estimated the mean and standard error of the neural responses from Fig 4 in [44]. Using these descriptive statistics, we calculated a t-value for the direct stimulus–brain comparison for all beat- and non-beat-related frequencies. Based on the critical t-value used in [44] (i.e., t_crit_ = 1.3968), we determined whether each stimulus–brain comparison reached statistical significance. We then quantified the number of times the two preprocessing pipelines led to conflicting conclusions.

**Fig 4:**
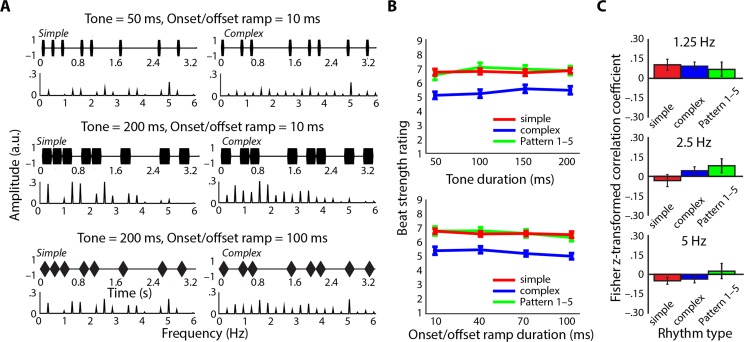
Beat perception does not depend on amplitudes at beat-related frequencies in stimulus spectra. **A.** Examples of simple (left column) and complex (right column) rhythms used in Experiment 2a shown together with amplitude spectra. Top: 50-ms tones with 10-ms onset/offset ramps; Middle: 200-ms tones with 10-ms onset/offset ramps; Bottom: 200-ms tones with 100-ms onset/offset ramps. Note the changes to the amplitude spectra that result from changing acoustic stimulus features even for the same onset pattern. **B.** Beat strength ratings did not change as a function of tone duration (top) or onset/offset ramp duration (bottom) despite their different amplitude spectra. Beat strength ratings did depend on onset pattern (i.e., whether the rhythm was simple or complex, shown in color). **C.** Beat strength ratings were not significantly correlated with amplitudes at beat-related frequencies 1.25 Hz (top), 2.5 Hz (middle), or 5 Hz (bottom) for any rhythm type (colors same as B). Fisher z-transformed correlation coefficients averaged across participants are shown with standard error of the mean and were not significantly different from zero.

### Results and discussion

The amplitudes at every beat-related frequency in the stimulus spectrum varied depending on both the duration of the tones making up the rhythms (Fig 2B, shown for 10-ms onset ramp only) and the duration of the linear onset/offset ramps gating each tone onset (Fig 2C, shown for 200-ms tones only; the results for all combinations of tone duration and onset/offset ramp duration are presented in the Supporting Information S1 Fig). Interestingly, the function relating amplitudes to tone duration and onset/offset ramp duration was different for each beat-related frequency. For example, while 1.25-Hz amplitude increased linearly with tone duration, 5-Hz amplitude was related to tone duration by a quadratic function, with peak amplitudes observed for tone durations of about 100 ms. Moreover, the precise function relating spectral amplitude to tone duration or onset/offset ramp duration also depended on the specific rhythm.

The implication is this: enhancement of neural responses at beat- and meter-related frequencies may be harder (easier) to observe when rhythms already have high (low) energy in the stimulus spectrum at beat- and meter-related frequencies simply because of the acoustic properties of the tones making up the rhythm–and not necessarily because of the strength of the beat percept. Take for example Pattern 1: XooXXXoXXXoX, where ‘X’ corresponds to a tone and ‘o’ to a silence. If this rhythm is composed of 50-ms tones with 10-ms onset/offset ramps, 1.25-Hz spectral amplitude is 0.105. If the same rhythm is composed of 200-ms tones with 10-ms onset/offset ramps, 1.25-Hz spectral power is 0.455 (Supporting Information S1 Fig). Thus, two rhythms with statistically identical perceived beat strengths (as demonstrated by our behavioral Experiment 2a) are characterized by a three-fold discrepancy in spectral amplitude at 1.25 Hz (a beat- and meter-related frequency). This discrepancy may make it difficult for a relative neural enhancement to be observed (in the case of high stimulus amplitude), or easier (in the case of low stimulus amplitude).

It is of course possible (as brought to our attention by a reviewer) neural spectra will mimic stimulus spectra, and thus are dependent on acoustic properties. Neural amplitudes at beat- and meter-related frequencies would then be enhanced over and above the relative amplitudes in the stimulus spectra. Our argument is that this neural enhancement may not be equally likely to be observed in two cases with a three-fold difference in spectral amplitude at a beat- and meter-related frequency based on the stimulus spectrum alone, and if true, neural enhancement (with respect to the stimulus spectrum) may not be a valid metric to infer beat perception.

One subtle but critical difference between the current stimulus analysis and that of [44] is that we used the standard Matlab *hilbert* function to generate stimulus envelopes, rather than the Hilbert transform as implemented in the MIR Toolbox, which applies a time-domain filter before applying the transformation (an IIR filter with a low-pass cutoff of 50 Hz; [56]). For this reason, the two techniques yielded different results in the frequency domain for identical stimuli (Fig 3), so we evaluated the consequences of comparing neural responses to stimulus envelopes calculated by the MIR toolbox versus the Matlab *Hilbert* function. We found that across all beat- and non-beat-related frequencies, the statistical outcomes disagreed for the two preprocessing pipelines 38.2% of the time. That is, based on the two methods for analyzing the stimuli, the stimulus–brain comparison led to the same statistical outcomes (in terms of significance and direction of that significance) for only 61.8% of comparisons. For 26.5% of comparisons, the two techniques disagreed in terms of statistical significance (one pipeline lead to significance while the other did not), and for 11.8% of comparisons the two pipelines lead to opposite, but statistically significant, conclusions regarding neural enhancement. We confirmed these results by inspecting only beat- and meter-related frequencies, and found that the results of the MIR-preprocessed were incorrect almost half of the time (45.4% of comparisons: 31.8% of all comparisons disagreed in terms of significance, and 13.6% led to significance in the wrong direction).

To summarize, frequency-domain representations of stimulus envelopes (i.e., stimulus spectra) are sensitive to acoustic features of the individual tones making up the rhythms (i.e., tone duration, onset/offset ramp duration), as well as the preprocessing stages used to obtain the envelope (i.e., time-domain filtering as implemented in the MIR Toolbox). In turn, direct comparisons between stimulus and brain, proposed to yield evidence for neural resonance at beat-related frequencies and a neural marker of beat perception [44], also depend on acoustic stimulus features. We caution against using this approach to infer the presence of neural resonance, because amplitudes at beat-related frequencies vary widely as a function of tone duration or onset/offset duration (Fig 2), but these acoustic stimulus features are not expected to impact beat perception. The behavioral experiments we report next directly address this claim.

## Experiment 2: Dissociating stimulus spectra from beat perception

We are arguing that directly comparing amplitudes at beat-related frequencies from stimulus spectra to those from neural spectra to infer the presence of neural resonance is unlikely to be valid. We have shown that amplitudes at beat-related frequencies in stimulus spectra vary depending on acoustic stimulus features, here the duration of the tones and onset/offset ramps. However, we have explicitly assumed that changes to these acoustic stimulus features do not affect beat perception, thus dissociating stimulus spectra from behavior. Here, we tested this assumption by having participants rate the perceived beat strength of either rhythms composed of different tone types, but whose perceived beat strength we expected to be the same (Experiment 2a) or rhythms with identical stimulus spectra, but whose perceived beat strength we expected to be different (Experiment 2b). We expected to observe a dissociation between stimulus spectra and perceived beat strength, which would further support our argument that comparing stimulus spectra directly to neural spectra will not inform us about the neural correlates of beat perception.

### General methods

#### Participants

18 individuals (11 female, mean age = 21.9 years, SD = 1.75 years) with self-reported normal hearing participated in Experiments 2a and 2b. Participants had between 0 and 25 years of musical training (M = 6.6 years, SD = 7.2 years), and 5 were currently practicing music. Because of a technical problem, two participants were not able to participate in Experiment 2a (making a final sample size of n = 16 for Experiment 2a and n = 18 for Experiment 2b). Written informed consent was obtained from each participant prior to participation. Participants were paid $10 for their time. The University of Western Ontario’s Non-Medical Research Ethics Board approved all procedures.

#### Procedure

Participants were presented with a single rhythm on each trial, and were asked to rate the strength of the perceived beat on a scale ranging from 1 (Very weak beat) to 9 (Very strong beat) using the number keys on the laptop keyboard. They were instructed to make their ratings based on how easily they thought they would be able to tap the beat (but were instructed not to move). We have favored asking participants to rate the perceived beat strength over, for example, asking participants to tap the beat along with each rhythm. Most importantly, given our interest in beat perception, and not beat production, asking participants to rate beat strength is preferable, as tapping actually *alter* beat perception, for example, strengthening the perceived beat in a complex rhythm [22]. Furthermore, finger-tapping data are often inherently difficult to interpret given different preferred tapping frequencies and some individuals’ inability to tap stably (e.g., [44], Fig 1). Using our rating measure, we’ve shown a robust difference between simple and complex rhythms, whose beat strengths we have validated using counterevidence scores (C-scores) taken from the Povel and Essens model [6]. We would thus like to suggest that our rating measure is a direct index of beat perception.

Stimuli were presented over Sennheiser HD 280 Pro headphones at a comfortable listening level to participants seated in a sound-attenuated booth. The Psychophysics Toolbox [57–59] for Matlab running on a Dell Precision M4600 laptop running Windows 7 Professional controlled stimulus presentation and response collection; universal ASIO sound driver was installed. The entire experiment took approximately one hour (Experiment 2a: 45 minutes, Experiment 2b: 5 minutes).

#### Effect size

We report effect size as r_equivalent_ (r_e_) for single-df tests and partial η^2^ (η^2^_p_) for main effects and interactions with more than one degree of freedom corresponding to the effect of interest [60].

### Experiment 2a: Methods

#### Stimuli

Stimuli were 35 unique rhythms of three types: a) 15 “simple” rhythms, b) 15 “complex” rhythms, and c) Pattern 1–5 from Experiment 1. Simple and complex rhythms were based on those used in previous work [61]. Simple rhythms were composed of intervals related by integer ratios (1:2:3:4), and had a regular grouping of intervals that resulted in event onsets always being present at “on-beat” locations given a quadruple meter (i.e., at every fourth location starting from the first location). Simple rhythms thus induced a relatively strong sense of a beat. Complex rhythms were also composed of intervals related by integer ratios, but intervals were grouped irregularly and thus did not induce a strong beat percept.

**Fig 5:**
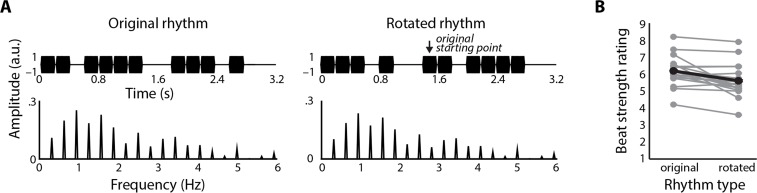
Beat perception differs for rhythms with identical frequency-domain representations. **A.** By rotating simple rhythms (i.e., playing them starting from a different point in the sequence), we created two version of rhythms with numerically identical frequency-domain representations. **B.** Beat strength ratings differed significantly between original and rotated rhythms. Individual participant data are shown in gray, and mean data are overlaid in black.

Simple rhythms were created by combining individual 4-unit measures, each of which contained between one and four intervals, as in [61]. The possible interval patterns in a measure were: 1-1-1-1, 1-1-2, 2-1-1, 1-2-1, 2–2, 1–3, 3–1, and 4. Measures occurred with probabilities estimated from [61]. Simple rhythms comprised 4 measures. Individual measures with interval structures 1–3 and 1-2-1 never occurred in the first two measures of the simple rhythm and no neighboring measures had the same interval structure. Complex rhythms were created by shuffling the individual intervals making up each simple rhythm (Fig 5A). For complex rhythms, events occurred at maximally 33% of on-beat locations (i.e., at every fourth position starting with the first). Moreover, no more than two “simple” measures could occur consecutively in the complex rhythm. Simple rhythms, complex rhythms, and Pattern 1–5 were looped twice so that they lasted 6.4 seconds (4.8 seconds for three Patterns 1, 3, and 4 from [44]). We confirmed differences in model-based estimates of beat strength between our simple and complex rhythms using counterevidence scores from the Povel & Essens model [6] calculated for the 6.4-s rhythms (simple: C = 0 ± 2; complex: C = 24 ± 2; Pattern 1–5: C = 0 ± 0.5; median ± interquartile range, IQR). Counterevidence scores quantify the degree to which a rhythm conflicts with a specific metrical interpretation, and can thus be interpreted as a measure of metric complexity. Low C-scores indicate simple rhythms, and high C-scores indicate complex rhythms. Simple rhythms and Pattern 1–5 did not differ with respect to counterevidence scores (Mann–Whitney U test: z = 0.49, p = .62, r_e_ = .12) but, as expected, both types of rhythms had significantly less counterevidence than complex rhythms (vs. simple: z = 3.50, p < .001, r_e_ = .76; vs. complex: z = 4.85, p < .001, r_e_ = .71).

All rhythms were presented at a base rate of 5 Hz (i.e., the shortest possible inter-onset interval was 200 ms). Each rhythm was presented 8 times throughout the experiment, taking on 4 tone durations (50, 100, 150, 200 ms; onset/offset ramp duration was fixed at 10 ms) and 4 onset/offset ramp durations (10, 40, 70, 100 ms; tone duration was fixed at 200 ms). Thus, each participant responded to a total of 280 rhythms (35 rhythms × 4 tone durations + 35 rhythms × 4 onset/offset ramp durations).

#### Data analysis

To assess the effects of acoustic stimulus features (and thus the resulting variations in stimulus spectra) on beat perception, we conducted two separate 3 (Rhythm type: simple, complex, Pattern 1–5) × 4 (Tone duration: 50, 100, 150, 200 ms; or Onset/offset ramp duration: 10, 40, 70, 100 ms) repeated-measures ANOVAs on beat-strength ratings. Degrees of freedom were adjusted according to the Greenhouse-Geiser correction when sphericity was violated. Moreover, we conducted a correlation analysis to further address whether the amplitudes at beat-related frequencies in the stimulus FFT would predict beat perception. Within each rhythm type category, separately for each participant, we calculated a Pearson correlation between the amplitudes at beat-related frequencies in the stimulus spectra (1.25, 2.5, and 5 Hz) with behavioral ratings of beat strength. In order to rule out the possibility that amplitudes at beat-related frequencies in the stimulus spectra predicted behavioral ratings of beat strength for a specific, fixed combination of acoustic features, we calculated the same correlations separately for every unique combination of tone duration and onset/offset ramp duration. Correlation coefficients were Fisher z-transformed and tested against zero across participants. False discovery rate (FDR) correction [62, 63] was applied to compensate for multiple comparisons (α_FDR_ = .05).

### Results and discussion

We predicted that beat strength ratings would depend on the rhythms’ onset structure (i.e., whether they were simple or complex), but would not depend on the acoustic features of the tones making up each rhythm. For the ANOVA testing the effects of tone duration (Fig 4B), we observed a significant main effect of Rhythm type (F(2,30) = 15.71, p < .001, η^2^_p_ = .51). Complex rhythms were rated as having a weaker beat than simple rhythms (t(15) = 4.44, p < .001, r_e_ = .75) and Pattern 1–5 (t(15) = 4.74, p < .001, r_e_ = .77). The latter two rhythm types did not differ from each other in terms of perceived beat strength (t(15) = –0.33, p = .75, r_e_ = .08). Neither the main effect of Tone duration (F(3,45) = 1.83, p .16, η^2^_p_ = .11) nor the Rhythm type × Tone duration interaction (F(3.37,50.60) = 1.51, p = .09, η^2^_p_ = .09) reached significance.

For the ANOVA testing the effects of onset/offset ramp duration (Fig 4B), the main effect of Rhythm type was significant (F(2,30) = 15.65, p < .001, η^2^_p_ = .51). Again, complex rhythms were rated as having a weaker beat than either simple rhythms (t(15) = 4.45, p < .001, r_e_ = .75) or Pattern 1–5 (t(15) = 4.70, p < .001, r_e_ = .77). The latter two rhythm types did not differ significantly from each other in terms of perceived beat strength (t(15) = –0.05, p = .97, r_e_ = .01). Neither the main effect of Onset/offset ramp duration (F(1.35,20.28) = 2.83, p = .10, η^2^_p_ = .16) nor the Rhythm type × Onset/offset ramp duration interaction (F(6,90) = 0.86, p = .53, η^2^_p_ = .05) reached significance.

Within each rhythm type, we also calculated correlations between beat strength ratings and the amplitudes at the beat-related frequencies (i.e., 1.25, 2.5, and 5 Hz) obtained from the stimulus spectra separately for each participant, and then tested the Fisher z-transformed correlation coefficients against zero. After correcting for multiple comparisons (9 tests), none of the correlations reached significance (adjusted p_FDR_ ≥ .07, Fig 5C), indicating that amplitudes of the peaks at beat-related frequencies in the stimulus spectra had no consistent relationship to ratings of beat strength. This was also true when we calculated the correlations for specific, fixed combinations of tone duration and onset/offset ramp duration (p_FDR_ ≥ .08).

Thus, although we changed the frequency-domain representations of the rhythms that participants heard (Fig 4A), the acoustic manipulations we used to bring about these changes did not affect perceived beat strength. Moreover, a more sensitive correlational test of the relation between stimulus amplitude peaks at beat-related frequencies and perceived beat strength also revealed numerically very small and nonsignificant correlations. Instead, perceived beat strength was strongly driven by the onset structure of the rhythms; simple rhythms (designed to have a strong sense of beat) were, as expected, perceived as having a significantly stronger beat than complex rhythms (designed to have a weak sense of beat).

### Experiment 2b: Methods

#### Stimuli

Stimuli were 15 4-measure simple rhythms (not the same set as Experiment 2a) plus Pattern 1–5 (from [44]), which were either 3 (Patterns 1, 3, 4) or 4 measures long (Patterns 2, 5). Based on the results of Experiment 2a, we treated simple rhythms and Pattern 1–5 as a single category. “Rotated” versions of each simple rhythm were created by shifting the starting position of the rhythm to a different tone. The new starting position for each rhythm was chosen to maximize counterevidence scores for the rotated versions of the rhythms [6]; original: C = 1 ± 1; rotated: C = 12 ± 1, median ± IQR; Wilcoxon sign-rank test: W = 120, p < .001, r_e_ = .76), and thus likely minimize perceived beat strength. We chose this manipulation because the FFT assumes circularity in the time-series data, which means that it is insensitive to the starting position within the rhythm. We thus tested whether two versions of the same rhythm (i.e., original and rotated) can be perceived differently in terms of beat strength despite having numerically identical stimulus spectra (Fig 5).

All rhythms were presented at a base rate of 5 Hz (i.e., the shortest possible inter-onset interval was 200 ms) and were composed of 990-Hz sine tones. Rhythms were not repeated; 3-measure rhythms lasted 2.4 s and 4-measure rhythms lasted 3.2 s. Each of the 40 unique rhythms (20 original, 20 rotated) was presented once. The experiment lasted approximately 5 minutes.

#### Data analysis

A paired-samples t-test was performed on beat strength ratings for original compared to rotated rhythm versions.

### Results and discussion

Beat strength ratings were significantly larger for original compared to rotated versions of the same rhythms (t(17) = 3.80, p = .001, r_e_ = .66, Fig 5). Thus, although the frequency-domain representations were numerically identical for the two rhythm types, the strength of the perceived beat differed between them.

## General discussion

In the current paper, we have demonstrated that 1) amplitudes at beat-related frequencies in the stimulus spectrum change depending on the acoustic stimulus features of the tones making up the rhythms (i.e., tone duration, onset/offset ramp duration); 2) these changes in the stimulus spectrum do not give rise to changes in beat perception; and 3) rhythms with identical stimulus spectra can nonetheless be perceived differently with respect to beat strength. The empirical results confirm a long-standing assumption in the literature that the acoustic features of tones making up the rhythm should have little consequence for beat perception. We hope that this work provides a convincing demonstration that the stimulus envelope and strength of the beat perception are dissociable, and thus caution should be exercised when using direct stimulus–brain comparisons to make inferences about beat perception.

Beat perception has been studied using myriad tone types to compose rhythm stimuli, for example, filled intervals and sine tones [19, 61]; empty intervals and sine tones [17, 64]; noise bursts [65], naturalistic drum sounds [18, 66]; woodblocks [67, 68]; musical pieces [12, 15]. Aggregating findings across the literature suggests that the acoustic features of the tones should have little to do with beat perception. Here, we systematically tested this by varying the tone duration and onset/offset ramp duration of sine tones composing simple and complex rhythms. As expected, simple rhythms were perceived as having a stronger beat than complex rhythms. Critically, manipulations of the acoustic features of tones did not affect perceived beat strength. Moreover, we generated a situation in which two rhythms (one that was simply a rotated version of the other) had numerically identical frequency-domain representations, but evoked different beat strength percepts. Overall, we have demonstrated that the strength of a perceived beat is not predictable from a rhythm’s stimulus spectrum.

Our stimulus analysis focused on the five rhythms used in [44]. In the cited study, participants listened to repeating three- or four-measure rhythms while EEG was measured. The rhythms were all “simple”, which we confirmed here using counterevidence scores [6], and were thus likely to induce a strong sense of beat. Frequency-domain representations of EEG responses to the rhythms (neural amplitude spectra) were compared directly to frequency-domain representations of the rhythms themselves (stimulus amplitude spectra). When the (z-scored) magnitude of the neural response at a particular frequency was found to exceed the (z-scored) magnitude of the stimulus peak at the same frequency, the authors concluded that that frequency was enhanced in the neural response. In turn, the authors [44]{Nozaradan, 2016 #1343} suggested that enhanced peaks at beat-related frequencies reflect entrainment of a nonlinear oscillator by a stimulus rhythm and provide empirical support for neural resonance as a substrate of beat perception [9].

In light of the positive response of the scientific community to this version of the frequency-tagging approach [35–40], we would like to suggest caution in the interpretation of these findings. Here, we showed that the shape of the frequency-domain representation of a rhythm, including the relative amplitudes at beat-related frequencies, changes with alterations to the acoustic properties of the tones making up the rhythm (Fig 2 and S1 Fig; here, tone duration and onset/offset ramp duration). This is because any changes to the time-domain envelope translate to changes in the frequency-domain representation [55]. For this reason, frequency-domain representations are also dependent on pre-processing steps applied to the time-domain envelope. For example, the built-in filtering applied by the MIR Toolbox [56] naturally led to different frequency-domain representations than the Matlab *hilbert* function applied without filtering. We estimated that, for beat- and meter-related frequencies, the method employed by the authors [44] would have resulted in incorrect statistical conclusions about half of the time. Taking these observations together, we suggest being cautious in the interpretation of direct frequency-domain stimulus–brain comparisons.

It is important to note that we are specifically arguing against the practice of directly comparing frequency-domain representations of stimulus rhythms and neural responses. We are not diminishing the practice of examining neural responses in the frequency domain, per se. Indeed, the demonstration of enhanced neural responses at a frequency corresponding to an *imagined* accent structure during listening to a metronome is extremely convincing regarding the utility of this approach [41]. Similarly, interactions between entrained auditory and motor responses during finger tapping to the beat have been demonstrated using a version of the frequency-tagging approach that does not entail direct stimulus–brain comparisons [16]. Moreover, we are not dismissing the hypothesis that neural resonance resulting from entrainment of a nonlinear oscillator(s) underlies beat perception [9, 23]. However, we would like to suggest that the way forward involves coupling EEG measures of entrainment to *behavior*, for example using 1) paradigms that provide a window into the advantages of picking up on regularity in nonisochronous stimuli (i.e., a “beat-based advantage”; [61]), or 2) interaction designs that can reveal differences between rhythm types (e.g., simple vs. complex; [61, 69]), participant groups (e.g., good vs. poor beat perceivers, musicians vs. nonmusicians, stutters vs. controls; [17, 70–72]), or cognitive demands (e.g., single- vs. dual-task; [73, 74]). Given the arguments outlined in the General Methods, we are envisioning work making use of non-finger-tapping behavioral paradigms.

To conclude, frequency-domain representations of rhythm envelopes depend strongly on acoustic features of the tones making up the rhythms (e.g., tone duration, onset/offset ramp duration) and preprocessing steps applied in the time domain (e.g., filtering). Moreover, changes to acoustic features and subsequent changes to amplitudes at beat-related frequencies in stimulus spectra do not affect modulations of perceived beat strength. Conversely, perceived beat strength can be different for rhythms with numerically identical frequency-domain representations. We emphasize caution in interpreting direct comparisons between stimulus rhythms and brain signals in the frequency domain, and suggest that a more fruitful approach to studying neural correlates of beat perception is to relate frequency-domain features of neural responses to rhythms to behavior and to examine modulations thereof under different experimental conditions.

## Supporting information

S1 Fig Frequency-domain amplitudes at beat-related frequencies plotted for all combinations of tone duration (y-axis) and onset/offset ramp duration (x-axis), shown separately for Pattern 1–5. (PDF)

## References

[1] Fitch WT. The biology and evolution of music: A comparative perspective. Cognition. 2006;100:173–215. 10.1016/j.cognition.2005.11.009 16412411

[2] Hagmann CE, Cook RG. Testing meter, rhythm, and tempo discriminations in pigeons. Behavioral processes. 2010;85:99–110. 10.1016/j.beproc.2010.06.015 20600695

[3] McDermott J, Hauser MD. Nonhuman primates prefer slow tempos but dislike music overall. Cognition. 2007;104:654–68. 10.1016/j.cognition.2006.07.011 16935277

[4] Large EW, Palmer C. Perceiving temporal regularity in music. Cognitive Science. 2002;26:1–37.

[5] Parncutt R. A perceptual model of pulse salience and metrical accents in musical rhythms. Music Perception. 1994;11:409–64.

[6] Povel DJ, Essens P. Perception of temporal patterns. Music Perception. 1985;2(4):411–40. 10.3758/bf03207132 3991313

[7] Fitch WT, Rosenfeld AJ. Perception and production of syncopated rhythms. Music Perception. 2007;25:43–58.

[8] Honing H. Without it no music: beat induction as a fundamental musical trait. Ann N Y Acad Sci. 2012;1252:85–91. 10.1111/j.1749-6632.2011.06402.x 22524344

[9] Large EW. Resonating to musical rhythm: Theory and experiment In: Grondin S, editor. Psychology of Time: Emerald Group Publishing Limited; 2008.

[10] Honing H, Bouwer FL, Háden GP. Perceiving temporal regularity in music: The role of auditory event-related potentials (ERPs) in probing beat perception In: Merchant H, de Lafuente V, editors. Neurobiology of Interval Timing. Advances in Experimental Medicine and Biology New York: Springer; 2014 p. 305–23. 10.1007/978-1-4939-1782-2_16 25358717

[11] Patel AD, Iversen JR, Chen Y, Repp BH. The influence of metricality and modality on synchronization with a beat. Exp Brain Res. 2005;163(2):226–38. 10.1007/s00221-004-2159-8 15654589

[12] Iversen JR, Patel AD, editors. The Beat Alignment Test (BAT): Surveying beat processing abilities in the general population. International Conference on Music Perception and Cognition (ICMPC 10); 2008; Sapporo, Japan.

[13] Farrugia N, Benoit C-E, Harding E, Kotz SA, Dalla Bella S, editors. BAASTA: Battery for the assessment of auditorz sensorimotor and timing abilities. International Conference on Music Perception and Cognition (ICMPC 12); 2012; Thessaloniki, Greece.

[14] Fujii S, Schlaug G. The Harvard Beat Assessment Test (H-BAT): A battery for assessing beat perception and production and their dissociation. Frontiers in Human Neuroscience. 2013;7. 10.3389/fnhum.2013.00771 PMC3840802 24324421

[15] Müllensiefen D, Gingras B, Musil J, Stewart L. The musicality of non-musicians: An index for assessing musical sophistication in the general population. PLoS ONE. 2014;9:e89642 10.1371/journal.pone.0089642 24586929 PMC3935919

[16] Nozaradan S, Zerouali Y, Peretz I, Mouraux A. Capturing with EEG the neural entrainment and coupling underlying sensorimotor syncrhonization to the beat. Cereb Cortex. 2015;25:736–47. 10.1093/cercor/bht261 24108804

[17] Grahn JA, McAuley JD. Neural bases of individual differences in beat perception. Neuroimage. 2009;47:1894–903. 10.1016/j.neuroimage.2009.04.039 19376241

[18] Winkler I, Háden GP, Ladinig O, Sziller I, Honing H. Newborn infants detect the beat in music. Proceedings of the New York Academy of Sciences USA. 2009;106:2468–71. 10.1073/pnas.0809035106 PMC2631079 19171894

[19] Grahn JA, Brett M. Impairment of beat-based rhythm discrimination in Parkinson's disease. Cortex. 2009;45:54–61. 10.1016/j.cortex.2008.01.005 19027895

[20] Patel AD, Iversen JR, Bregman MR, Schulz I. Experimental evidence for synchronization to a musical beat in a nonhuman animal. Curr Biol. 2009;19:827–30. 10.1016/j.cub.2009.03.038 19409790

[21] Merchant H, Grahn JA, Trainor LJ, Rohrmeier M, Fitch WT. Finding the beat: A neural perspective across humans and non-human primates. Philosophical Transactions of the Royal Society B. 2015;370:20140093. 10.1098/rstb.2014.0093 PMC4321134 25646516

[22] Su Y-H, Pöppel E. Body movement enhances the extraction of temporal structures in auditory sequences. Psychol Res. 2012;76:373–82. 10.1007/s00426-011-0346-3 21695472

[23] Large EW, Snyder AC. Pulse and meter as neural resonance. Ann N Y Acad Sci. 2009;1169:46–57. 10.1111/j.1749-6632.2009.04550.x 19673754

[24] Buzsaki G, Draguhn A. Neuronal oscillations in cortical networks. Science. 2004;25:1926–9. 10.1126/science.1099745 15218136

[25] Bishop GH. Cyclic changes in the excitability of the optic pathway of the rabbit. Am J Physiol. 1933;103:213–24.

[26] Lakatos P, Karmos G, Mehta AD, Ulbert I, Schroeder CE. Entrainment of neuronal oscillations as a mechanism of attentional selection. Science. 2008;320:110–3. 10.1126/science.1154735 18388295

[27] Kayser C, Wilson C, Safaai H, Sakata S, Panzeri S. Rhythmic auditory cortex activity at multiple timescales shapes stimulus–response gain and background firing. The Journal of Neuroscience. 2015;35:7750–62. 10.1523/JNEUROSCI.0268-15.2015 25995464 PMC4438125

[28] Kayser C, Montemurro MA, Logothetis NK, Panzeri S. Spike-phase coding boosts and stabilizes information carried by spatial and temporal spike patterns. Neuron. 2009;61:597–608. 10.1016/j.neuron.2009.01.008 19249279

[29] Thut G, Schyns P, Gross J. Entrainment of perceptually relevant brain oscillations by non-invasive rhythmic stimulation of the human brain. Frontiers in Psychology. 2011;2. 10.3389/fpsyg.2011.00170 PMC3142861 21811485

[30] Henry MJ, Obleser J. Frequency modulation entrains slow neural oscillations and optimizes human listening behavior. Proceedings of the National Academy of Sciences USA. 2012;109:20095–100. 10.1073/pnas.1213390109 PMC3523826 23151506

[31] Herrmann B, Henry MJ, Grigutsch M, Obleser J. Oscillatory phase dynamics in neural entrainment underpin illusory percepts of time. The Journal of Neuroscience. 2013;33:15799–809. 10.1523/JNEUROSCI.1434-13.2013 24089487 PMC6618472

[32] Lakatos P, Musacchia G, O'Connell MN, Falcher AY, Javitt DC, Schroeder CE. The spectrotemporal filter mechanism of auditory selective attention. Neuron. 2013;77:750–61. 10.1016/j.neuron.2012.11.034 23439126 PMC3583016

[33] Fujioka T, Trainor LJ, Large EW, Ross B. Internalized timing of isochronous sounds is represented in neuromagnetic Beta oscillations. The Journal of Neuroscience. 2012;32:1791–802. 10.1523/JNEUROSCI.4107-11.2012 22302818 PMC6703342

[34] Snyder JS, Large EW. Gamma-band activity reflects the metric structure of rhythmic tone sequences. Cognitive Brain Research. 2005;24:117–26. 10.1016/j.cogbrainres.2004.12.014 15922164

[35] Keller PE, Nozaradan S. Neural entrainment during musical rhythm perception is correlated with individual differences in temporal prediction during sensorimotor synchronization. Frontiers in Human Neuroscience Conference Abstract: XII International Conference on Cognitive Neuroscience (ICON–XII). 2015.

[36] Nozaradan S, Jonas J, Vignal JP, Maillar L, Mouraux A, editors. Neural entrainment to musical rhythms in the human auditory cortex, as revealed by intracerebral recordings. 30th International Conference of Clinical Neurophysiology (ICCN) of the IFCN; 2014; Berlin, Germany.

[37] Rajendran V, Garcia-Lazaro J, Lesica N, Schnupp JWH. Musical gerbils. 9th Federation of European Neuroscience Societies Forum of Neuroscience; Milan, Italy2014.

[38] Nozaradan S, Jonas J, Vignal JP, Maillard L, Mouraux A. Neural entrainment to musical rhythms in human auditory cortex, as revealed by intracerebral recordings. Program No 21407 Neuroscience Meeting Planner; San Diego, CA: Society for Neuroscience; 2013.

[39] Schlichting N. In time with rhythms–Beat perception and sensorimotor synchronization investigated with EEG. MEi:CogSci Conference 2015; Ljubljana2015.

[40] Rajendran V, Garcia-Lazaro J, Lesica N, Schnupp JWH, editors. Neuronal entrainment to rhythm in the gerbil inferior colliculus. 5th International Conference on Auditory Cortex; 2014; Magdeburg, Germany.

[41] Nozaradan S, Peretz I, Missal M, Mouraux A. Tagging the neuronal entrainment to beat and meter. The Journal of Neuroscience. 2011;31:10234–40. 10.1523/JNEUROSCI.0411-11.2011 21753000 PMC6623069

[42] Chemin B, Mouraux A, Nozaradan S. Body movement selectively shapes the neural respresetnation of musical rhythms. Psychol Sci. 2014;25:2147–59. 10.1177/0956797614551161 25344346

[43] Nozaradan S. Exploring how musical rhythm entrains brain activity with electroencephalogram frequency-tagging. Philisophical Transactions of the Royal Society B. 2014;369:1–10. 10.1098/rstb.2013.0393 PMC4240960 25385771

[44] Nozaradan S, Peretz I, Mouraux A. Selective neuronal entrainment to the beat and meter embedded in a musical rhythm. The Journal of Neuroscience. 2012;32:17572–81. 10.1523/JNEUROSCI.3203-12.2012 23223281 PMC6621650

[45] Nozaradan S, Mouraux A, Jonas J, Colnat-Coulbois S, Rossion B, Maillard L. Intracerebral evidence of rhythm transform in the human auditory cortex. Brain Struct Funct. 2016. 10.1007/s00429-016-1348-0 27990557

[46] Giraud A-L, Poeppel D. Cortical oscillations and speech processing: Emerging computational principles and operations. Nat Neurosci. 2012;15:511–7. 10.1038/nn.3063 22426255 PMC4461038

[47] Peelle JE, Gross J, Davis MH. Phase-locked responses to speech in human auditory cortex are enhanced during comprehension. Cereb Cortex. 2013. 10.1093/cercor/bhs118 PMC3643716 22610394

[48] Cummins F. Oscillations and syllables: A cautionary note. Frontiers in Psychology. 2012;3. 10.3389/fpsyg.2012.00364 PMC3464477 23060833

[49] Peelle JE, Davis MH. Neural oscillations carry speech rhythm through to comprehension. Frontiers in Psychology. 2012;3:320 10.3389/fpsyg.2012.00320 22973251 PMC3434440

[50] Ding N, Simon JZ. Adaptive temporal encoding leads to a background-insensitive cortical representation of speech. The Journal of Neuroscience. 2013;27:5728–35. 10.1523/JNEUROSCI.5297-12.2013 PMC3643795 23536086

[51] Ding N, Simon JZ. Neural coding of continuous speech in auditory cortex during monaural and dichotic listening J Neurophysiol. 2012;107:78–89. 10.1152/jn.00297.2011 21975452 PMC3570829

[52] Ghitza O. Linking speech perception and neurophysiology: Speech decoding guided by cascaded oscillators locked to the input rhythm. Frontiers in Psychology. 2011;2. 10.3389/fpsyg.2011.00130 PMC3127251 21743809

[53] Pasley BN, David SV, Mesgarani N, Flinker A, Shamma SA, Crone NE, et al Reconstructing speech from human auditory cortex. PLoS Biol. 2012;10:e1001251 10.1371/journal.pbio.1001251 22303281 PMC3269422

[54] Mesgarani N, David SV, Fritz JB, Shamma SA. Influence of context and behavior on stimulus reconstruction from neural acitivity in primary auditory cortex. J Neurophysiol. 2009;102:3329–39. 10.1152/jn.91128.2008 19759321 PMC2804432

[55] Smith SW. Fourier Transform Properties The scientist and engineer's guide to digital signal processing. San Diego, CA: California Technical Publishing; 1997 p. 185–208.

[56] Larillot O, Toiviainen P, editors. A Matlab toolbox for musical feature extraction from audio. Proceedings of the 10th International Conference on Digital Audio Effects; 2007 September 10–15; Bordeaux, France.

[57] Brainard DH. The Psychophysics Toolbox. Spat Vision. 1997;10:433–6. 9176952

[58] Pelli DG. The VideoToolbox software for visual psychophysics: Transforming numbers into movies. Spat Vision. 1997;10:437–42. 9176953

[59] Kleiner M, Brainard DH, Pelli DG. What's new in Psychtoolbox-3? Perception. 2007;36:ECVP Abstract Supplement.

[60] Rosenthal R, Rubin DB. r(equivalent): A simple effect size indicator. Psychol Methods. 2003;8:492–6. 10.1037/1082-989X.8.4.492 14664684

[61] Grahn JA, Brett M. Rhythm and beat perception in motor areas of the brain. J Cogn Neurosci. 2007;19(5):893–906. 10.1162/jocn.2007.19.5.893 17488212

[62] Benjamini Y, Hochberg Y. Controlling the false discovery rate: A practical and powerful approach to multiple testing. Journal of the Royal Statistical Society Series B (Methodological). 1995;57:289–300.

[63] Benjamini Y, Yekutieli D. The control of the false discovery rate in multiple testing under dependency. The Annals of Statistics. 2001;29:1165–88.

[64] Grahn JA, Henry MJ, McAuley JD. FMRI investigation of cross-model interactions in beat perception: Audition primes vision, but not vice versa. Neuroimage. 2011;54:1231–43. 10.1016/j.neuroimage.2010.09.033 20858544 PMC3002396

[65] Motz BA, Erickson MA, Hetrick WP. To the beat of your own drum: Cortical regularization of non-integer ratio rhythms toward metrical patterns. Brain Cogn. 2013;81:329–36. 10.1016/j.bandc.2013.01.005 23434916 PMC3683872

[66] Ladinig O, Honing H, Háden GP, Winkler I. Probing attentive and preattentive emergent meter in adult listeners without extensive musical training. Music Perception 2009;26:377–86.

[67] Chen JL, Penhune VB, Zatorre RJ. Moving on time: Brain network for auditory-motor synchronization is modulated by rhythm complexity and musical training. J Cogn Neurosci. 2008;20:226–39. 10.1162/jocn.2008.20018 18275331

[68] Manning F, Schutz M. "Moving to the beat" improves timing perception. Psychon Bull Rev. 2013;20:1133–9. 10.3758/s13423-013-0439-7 23670284

[69] Lewis PA, Wing AM, Pope PA, Praamstra P, Miall RC. Brain activity correlates differentially with increasing complexity of rhythms during initialisation, synchronisation, and continuation phases of paced finger tapping. Neuropsychologia. 2004;42:1301–12. 10.1016/j.neuropsychologia.2004.03.001 15193939

[70] Nespoli G, Ammirante P, Russo FA. Musicians show enhanced neural synchronization at multiple timescales. Journal of the Canadian Acoustical Association. 2014;42:74–5.

[71] Wieland EA, McAuley JD, Dilley LC, Chang S-E. Evidence for a rhythm perception deficit in children who stutter. Brain Lang. 2015;144:26–34. 10.1016/j.bandl.2015.03.008 25880903 PMC5382013

[72] Etchell AC, Johnson BW, Sowman PF. Behavioral and multimodal neuroimaging evidence for a deficit in brain timing networks in stuttering: A hypothesis and theory. Frontiers in Human Neuroscience. 2014;8:467 10.3389/fnhum.2014.00467 25009487 PMC4070061

[73] Meltzer B, Reichenbach CS, Braiman C, Schiff ND, Hudspeth AJ, Reichenbach T. The steady-state response of the cerebral cortex to the beaet of music reflects both the comprehension of music and attention. Frontiers in Human Neuroscience. 2015;9. 10.3389/fnhum.2015.00436 PMC4526810 26300760

[74] Gibbings AWC. How attention and beat perception modulate neural entrainment to rhythm. University of Western Ontario—Electronic Thesis and Dissertation Repository. 2014;Paper 2296.

