# Supplementary material for: What can we learn about beat perception by comparing brain signals and stimulus envelopes?
Source: PLoS One. 2017 Feb 22;12(2):e0172454. doi: 10.1371/journal.pone.0172454 (PMC5321456; doi:10.1371/journal.pone.0172454)
Supplement: S1 Fig — (PDF) [file pone.0172454.s001.pdf]

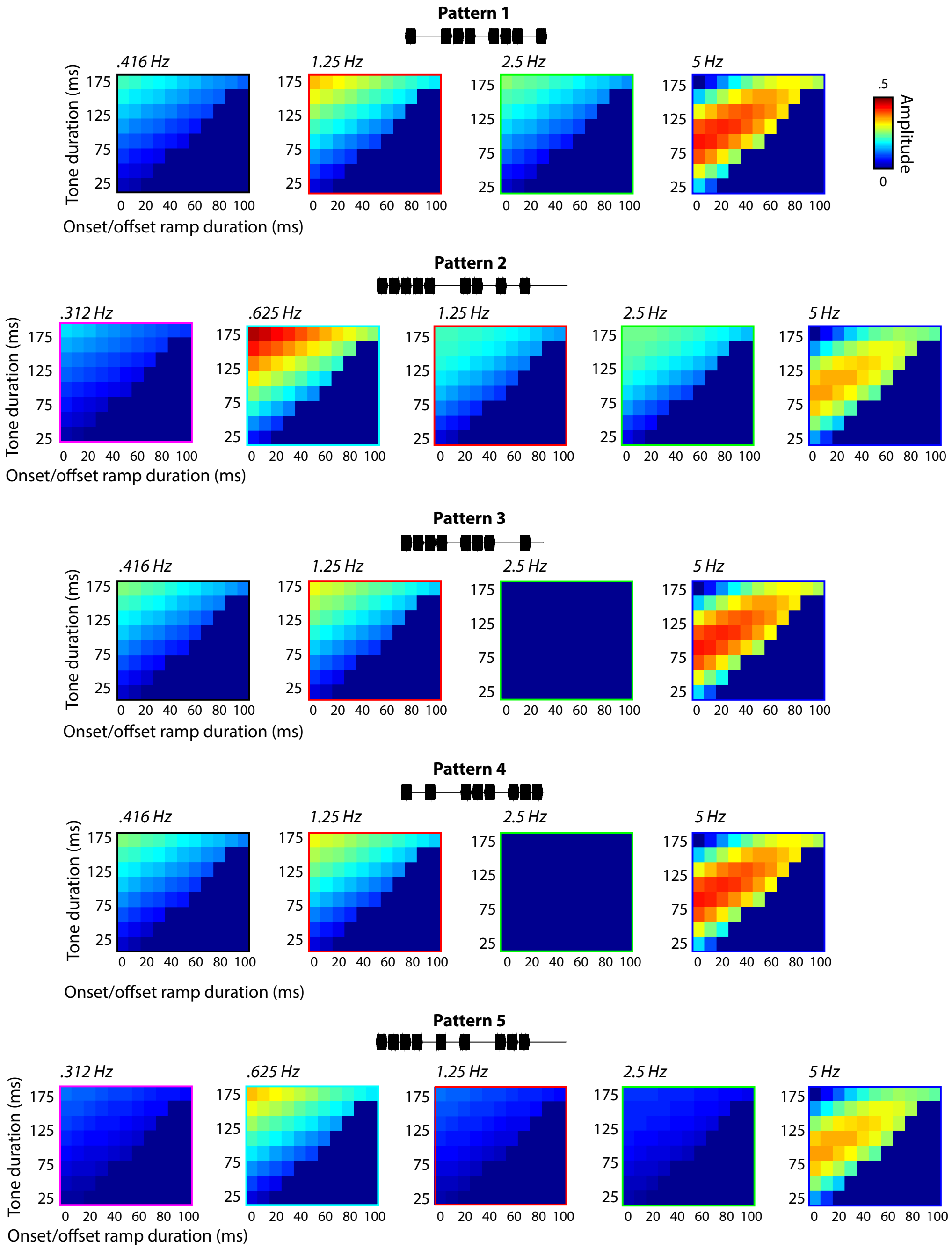

S1 Figure. Frequency-domain amplitudes at beat-related frequencies plotted for all combinations of tone duration (y-axis) and onset/offset ramp duration (x-axis), shown separately for Pattern 1–5. Colored boxes bounding each plot correspond to key in Fig. 2. Color bar (top right) is the same for all patterns.
